# WEAMR — A Weighted Energy Aware Multipath Reliable Routing Mechanism for Hotline-Based WSNs

**DOI:** 10.3390/s130506295

**Published:** 2013-05-13

**Authors:** Ali Tufail, Arslan Qamar, Adil Mehmood Khan, Waleed Akram Baig, Ki-Hyung Kim

**Affiliations:** Graduate School of Information and Communication, Ajou University, Suwon 443-749, Korea; E-Mails: alitufail@ajou.ac.kr (A.T.); arslanqamar@ajou.ac.kr (A.Q.); amtareen@ajou.ac.kr (A.M.K.); waleedbaig1@gmail.com (W.A.B.)

**Keywords:** wireless sensor network (WSN), sensor nodes, reliability, backbone routers

## Abstract

Reliable source to sink communication is the most important factor for an efficient routing protocol especially in domains of military, healthcare and disaster recovery applications. We present weighted energy aware multipath reliable routing (WEAMR), a novel energy aware multipath routing protocol which utilizes hotline-assisted routing to meet such requirements for mission critical applications. The protocol reduces the number of average hops from source to destination and provides unmatched reliability as compared to well known reactive *ad hoc* protocols *i.e.*, AODV and AOMDV. Our protocol makes efficient use of network paths based on weighted cost calculation and intelligently selects the best possible paths for data transmissions. The path cost calculation considers end to end number of hops, latency and minimum energy node value in the path. In case of path failure path recalculation is done efficiently with minimum latency and control packets overhead. Our evaluation shows that our proposal provides better end-to-end delivery with less routing overhead and higher packet delivery success ratio compared to AODV and AOMDV. The use of multipath also increases overall life time of WSN network using optimum energy available paths between sender and receiver in WDNs.

## 1. Introduction

Recent advances in the field of microelectronics and communications have brought the domain of Wireless Sensor Networks (WSNs) under the spotlight. WSNs can be formed by distributing inexpensive sensor devices on a large scale, typically, in a harsh environment. These sensor devices possess strict limitations in terms of memory, processing, battery and communication capabilities. However, WSNs hold the potential to be used in a wide array of crucial applications like industrial automation, chemical pollution monitoring, habitat monitoring, military, and healthcare, security and weather reporting [1]. Most of the sensors used in these applications are powered by batteries as energy source and these networks are expected to run for months without recharging.

**Figure 1.**
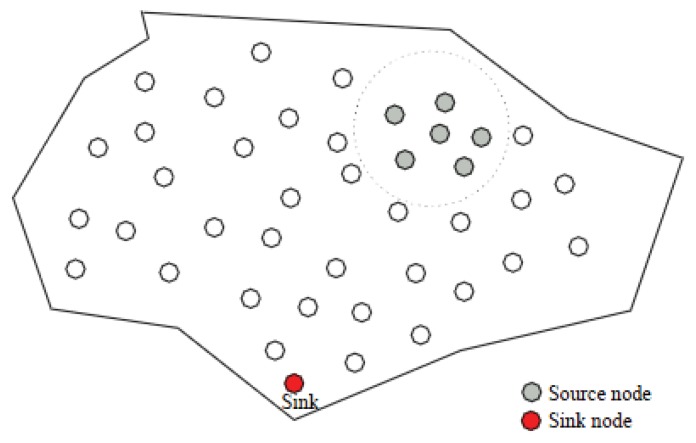
WSN with sink node.

Sensor nodes are involved in collecting readings or measurement of interest from the environment. The collected measurements are then required to be transmitted over to some external node, usually named as the sink node. In order to get the timely and correct analysis of the measurement the source to sink communication link has to be reliable. Typically, the source to sink communication is multi-hop and unreliable. Figure 1 shows a typical WSN architecture with a sink node:

A typical sensor node has to receive/sense data, process it or forward it to next hop in the sensor network. Reducing the amount of communication for path discovery to destination node and load balancing on multiple paths to reach the destination is very important to extend the life time of a sensor network. All of these unique characteristics of a WSN make it different from the traditional *ad hoc* network, therefore, WSNs require tailor-made protocols. Much of the research, that is currently underway, focuses on routing protocols with an emphasis on enhancing the lifetime of the network, *i.e.*, energy aware routing. Nevertheless, there are a number of crucial applications (like in military and healthcare) that not only require energy aware routing but also reliable routing. The inherent characteristics of WSNs, especially the wireless nature of communication, presents a major challenge towards development of such a protocol.

A new category of on-demand routing protocols (e.g., AODV [1], TORA [2], DSR [3]) for mobile *ad hoc* networks has been developed with a major focus on reducing routing overhead. These protocols work on the principle of reactive routing. In fixed topology networks, the protocols do not care much about routing protocol overhead such as: DSDV [4]. Such protocols are called proactive protocols. These protocols maintain all routes regardless of their feasibility in routing traffic. Such types of protocols do not suit networks like WSNs due to the limited energy and processing constraints of the sensor nodes. Therefore, a lot of research for routing in WSN networks is done in the domain of reactive/on-demand routing protocols. The key principle of an on-demand protocol is that whenever a source node wants to transmit data, it checks its routing table. If it knows where the destination, sink node or the gateway node is located it will transmit the data following a normal unicast routing pattern. If the destination node is unknown then the sender of the data initiates route discovery process by sending a route request. This is usually done using broadcast flooding. The sender node then waits for the request reply. Once the reply is received the sender node transmits the data using unicast. Since flooding in a network causes latency and overhead, such type of protocols are only suitable for small networks. In a large network, high latency and overhead can deem such protocols unworkable.

The earlier work in the domain of on demand routing considered only single path for route discovery and transmission. Since the same path is used over and over for future data transmissions, use of such a protocol in WSNs results in faster depletion of the energy of nodes that are in the route path. Thus such type of strategy can easily cripple a segment of a network and possibly make it inaccessible. To overcome this problem and to increase the reliability and lifetime of the network various multipath routing protocols have been developed. An on-demand multipath protocol discovers multiple paths in a single route discovery request and later can utilize each one of these if the best link fails. As a result next route discovery is only needed when all of the previous discovered routes fail. This helps in increasing the lifetime and reliability of the network with fewer interruptions. Moreover, end-to-end latency is reduced at the cost of minimal memory overhead at each routing node in the routing path.

In this paper, we have developed a new on-demand multipath routing protocol called WEAMR using a hotline-based architecture. WEAMR is based on a well-known and well accepted protocol AODV. WEAMR extends the concept of AODV to use multiple paths between source and destination nodes. The multiple path discoveries ensure loop free and disjoint routing paths. In this architecture, the gateway nodes are connected with highly reliable hotline links. The use of hotlines reduces the number of average hops between a source and a destination and provides better reliability as compared to traditional WSN routing [5]. WEAMR takes into account energy depletion criteria and switches to a less costly alternate routing path in order to increase the life time of a network. A route cost calculation mechanism has been proposed. This cost calculation mechanism does not require additional control packets.

The rest of the paper is organized as follows: Section 2 reviews the AODV protocol and hotline-based topology. In Section 3 we discuss related work. In Section 4 we introduce the WEAMR routing protocol. In Section 5 we compare the performance of our protocol with AODV using ns-3 simulations. Section 6 concludes this paper with our future work.

## 2. Ad hoc On-Demand Distance Vector Routing and Hotline Based Routing

AODV is a loop free single path reactive routing protocol for ad hoc networks. It is based on concepts of on-demand route discovery and destination sequence number from DSR [3] and DSDV [4]. AODV differs from DSR due to its hop by hop routing approach nature instead of source node routing. The following are some features of AODV that are required by WEAMR for its implementation.

### 2.1. Route Discovery and Maintenance in AODV

In reactive routing protocols, sending node is responsible for doing a route discovery process before it can send data to an undiscovered destination node. If the route path is already known the route discovery process is not required. The route discovery is an energy intensive operation as it depends on flooding of RREQ (Route – Request) packets. The sender, after sending the RREQ packets, waits for an expected RREP (Route – Reply) message which contains the hop by hop path towards the destination node. On receiving a RREQ packet, a reverse path by the node to the source node is set up using previous hop information in RREQ packet. This information is used for sending data backwards from the destination to the sender such as (acks, requests, *etc*.). If the intermediate node has a valid route path available for the destination, it replies back to the sender using unicast on the reverse path with a RREP packet which contains the full path to the destination. If the intermediate node does not have the route path information about destination node, it rebroadcasts the RREQ packet by updating the previous hop information with its own address. Duplication of RREQ is controlled by sequence number, so each intermediate node only forwards the RREQ message once. When the first RREQ message arrives at the destination node, it replies back with a RREP message using the reverse path information in the received RREQ packet. RREP packet travels back on the same path from which it arrived. As the RREP packet travels towards the source node it establishes a forward path towards the destination node [1]. In case of route failure RERR packets are used. The link failure detection and recovery mechanisms at the mac level can be used to detect the path failure efficiently. RERR packet is sent to all the source nodes that were using the intermediate node for reaching their destinations. All such paths are purged for use and source nodes need to reinitiate the route discovery process in order to communicate with destination nodes. Timer based mechanisms at each node helps in cleaning up the stale routes.

### 2.2. AODV is Loop Free

AODV keeps the routing paths loop free by using sequence numbers. Every destination has a monotonically increasing sequence number which is known as destination sequence number. Intermediate nodes with higher sequence number have more recent route information to the destination node and are given preference in case of multiple RREP messages. The AODV protocol prevents routing loops by maintaining an invariant that destination sequence numbers along any valid route [1].

### 2.3. Hotline-Based Reliable Routing

Recent research in WSNs to increase their reliability and life time, has given birth to many new and efficient ideas, and one such idea is the hotline-based reliable routing mechanism. It is well suited for real-time applications such as intrusion detection and battlefield surveillance applications, *etc.* The best case assumption for such a network is that the event should be detected with the help of just one packet transmission. To make such a packet driven reliability we use the concept of high-reliability hotline links between sensor gateways. In a large WSN the numbers of gateways is far less than the number of nodes, by using the concept of hotline-connected gateways, reliable and energy efficient routing in WSNs is possible [5].

## 3. Related Work

### 3.1. Existing Routing Protocols

Since wireless sensor networks and traditional wireless networks have different characteristics, a number of routing protocols have been proposed to address the challenges of sensor networks. Flat routing protocols are designed for networks with homogenous nodes, *i.e.*, all the network nodes have the same processing and data transmission capabilities, while their packet forwarding role is also similar. If a single path is used in transmission of data from sensor nodes to sink nodes use of such a protocol in WSN results in faster depletion of energy on nodes that are in the route path. Thus such type of strategy can easily cripple a segment of a network and possibly make it inaccessible. Discovering a path in a network is a resource intensive operation and to reduce overhead for path discovery on-demand routing protocols (e.g., AODV [1], TORA [2], DSR [3]) have been developed. These routing protocols are reactive routing protocols but they do not possess the capability to discover multiple paths, hence all of them suffer from the problem of non-uniform network usage. In [6,7] authors have proposed load sharing and network congestion reduction techniques which can be applied to any routing algorithm for WSNs domains. Directed Diffusion [8], Sensor Protocols for Information via Negotiation (SPIN) [9], Rumor Routing [10], Minimum Cost Forwarding Algorithm (MCFA) [11], and Energy-Aware Routing (EAR) [12] are some of the routing protocols that possess the capability of low topology maintenance cost and use multipath discovery to improve performance and reliability. Another protocol, AOMDV [13], which is a multipath routing protocol and an extension of AODV solves the issue of single path routing, but it doesn't take energy of the network into account. Another protocol energy efficient and collision aware (EECA) [14] is a multipath routing protocol which takes energy of nodes into account but this protocols assumes that the location and direction of nodes is well known in the network, also it tries to avoid collision by choosing distant route paths which we think is not a better approach as collision can be avoided at MAC level by RTS/CTS features [15].

All the protocols mentioned above try to solve the single path and energy constraints issues but according to our knowledge none of the protocols try to reduce number of hops and end to end latency using hotline based approach in wireless sensor networks, which provides higher throughout and reliable communication that is a key factor for mission critical WSN applications.

### 3.2. Multiple Gateways in WSNs

Generally a large number of nodes deployed in WSN are located in close proximity therefore interference and various other issues exist. The issues can be reduced by deploying gateways intelligently as proposed in [16]. The authors in [16] show that their proposed architecture provides better communication in a tradeoff with additional hardware cost. In [17], an intelligent estimation approach is proposed to calculate minimum number of gateways required to fulfill certain data latency related requirements for a WSN. Physical positioning of a gateway is vital for operation of a network and an efficient scheme to layout the gateways has been proposed in [18], where the authors show that the location of gateways has a marked influence on the data rate and overall power efficiency of the network.

### 3.3. Backbone Approaches for WSNs

The main barrier in deployment of WSNs is the limited energy at nodes and to overcome this various backbone approaches have been proposed in [19–21]. In [19] the authors prove that using a backbone can prolong network lifetime, reliability and scalability, whereas in [20] the authors present Sensor DMAC to reduce overhead of node selection, backbone formation and maintenance. The backbone is easily configurable with very less overhead. In [21], its authors propose a tree-based algorithm that maintains the structure of multihop clusters to form a stable backbone. The authors have tried to achieve load balancing and lower energy consumption using this approach.

### 3.4. Reliability in WSNs

Various studies [22–25] have been done in reliability analysis of homogeneous WSNs, but reliability analysis of backbone enabled WSNs is an unexplored domain by researchers so far. In [26] the authors have introduced the concept of hotlines to improve the reliability for WSNs. They have proved analytically that use of hotlines noticeably increases the reliability in WSNs. Most of the previous work, especially in the backbone approach area, focuses on energy efficiency in WSNs whereas we have combined versatile concepts, like energy aware routing, energy aware reactive routing, multipath routing and hotlines, to simultaneously enhance both reliability and energy efficiency in a WSN. Moreover, our paper suggests a brand new concept of WEAMR for WSNs to support the end-to-end delay requirements of different applications.

## 4. WEAMR Overview

WEAMR is an AODV-based reactive routing protocol designed to run in a cluster-based WSN. The protocol utilizes the additional benefits of hotline-enabled gateway nodes to increase the reliability and lifetime of the overall network. WEAMR is well suited for time critical applications in the WSN domain whereas a typical WSN topology with various nodes arranged to communicate in a hop by hop fashion cannot guarantee high reliability.

In a typical WSN, routing from source node to destination node is based on multiple hops. Several intermediate nodes working as relaying nodes forward the packet until reaches the destination. WSNs have inherently high link error rates. With multi-hop routing, the cumulative probability of error increases exponentially with the increasing number of hops [5]. Reliability is therefore a major concern in such networks. The use of hotlines between gateway nodes reduces the average hops between source and destination and thus increasing the reliability and energy efficiency of overall network.

### 4.1. Assumptions

**Figure 2.**
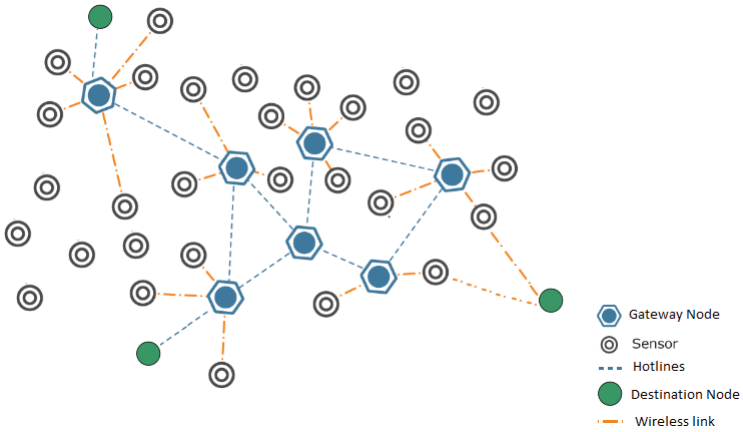
WSN with Hotlines Architecture.

We assume a two-tiered heterogeneous network. At first level, we have typical sensor nodes with constraints of energy and low power transmission. All the nodes are randomly distributed in a two dimensional grid. At second level, sensor gateways are present. These gateway nodes act as a cluster head. Moreover, the gateway nodes control the traffic flow and manage sensor motes deployed in different geographical locations. Gateway nodes are not energy constrained devices and are connected with each other in a bus topology. In traditional networks the gateways are connected with long haul wireless links but for this protocol the gateways are connected with high speed reliable links such as Ethernet or point-to-point wireless. We now introduce the important components of a hotline-based reliable WSN topology. Different mechanisms, like dividing the network into clusters, gateway association/dissociation and node-node, node-gateway and gateway-gateway communication will be described in detail in the following sub-sections. In traditional WSNs routing the expected path of end-to-end communication would include multiple hops. Packet delivery is dependent on several intermediate relaying sensor nodes. These sensor nodes forward the packets until they reach their destination. WSNs have inherently high link error rates. With multi-hop routing, the cumulative probability of error increases exponentially with the increasing number of hops. Thus the chances of failure are likely to increase with every additional hop. Reliability is therefore a main concern for mission-critical WSN deployments. This topology addresses the reliability issue by using the concept of hotlines between gateways. Hotlines reduce the number of average hops between a source and destination and provide better reliability as compared to traditional WSN routing. Our network topology is static for both sensor and gateway nodes. Figure 2 shows the overall network architecture:

We describe the topology and WEAMR in the following sections.

### 4.2. Network Clusters

Due to the availability of gateways, we can efficiently organize and manage the sensor nodes in a network. Each node would have to associate to a particular gateway making it the default gateway. All the nodes associated to one gateway form one cluster. Please note that gateway and cluster head are the same name used interchangeably. One of the important benefits of the clustering approach is to facilitate efficient and reliable communication using gateway hotlines. For example, one set of nodes is sensing the environment to get the data and then that data is being sent to another set of nodes or a sink for further computation. The default gateways can provide a fast and reliable routing mechanism to communicate this data. Nodes become the part of a cluster depending upon their hop count from the gateway. The nodes would get associated to any gateway by using the information in the Router Advertisement (RA) message. The routers would send router advertisement (RA) messages to help the nodes to associate to a particular gateway. When a sender node receives RA messages from several routers, it would differentiate the messages with help of current time to live (Cur TTL) field.

### 4.3. Internode Communications

**Figure 3.**
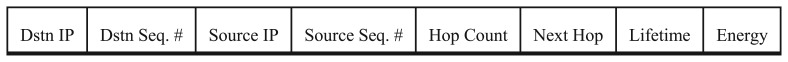
Proposed RREQ message format.

We have divided the network into different clusters and each cluster is associated with a default gateway, then routing of packets and route discovery is done within the cluster using RREQs. Our approach of intra-cluster routing is closer to the approach suggested in [13] (we recommend OSPF for gateway-to-gateway routing, as will be explained in Section 3.4). All the traffic (inter/intra-cluster and to/from the Internet) is routed through the gateway. The only exception here is the border nodes which are discussed later. Instead of an end-to-end wireless path, a packet is now routed through wireless-wired-wireless path, where the wired path is the communication between gateways via hotline and the wireless paths are used for intra-cluster communication. This approach clearly enhances the reliability of an end-to-end transmission. Under traditional routing, the path discovery process will discover an end-to-end wireless path. This path is less reliable, as shown in Figure 3, because the packet will traverse many wireless hops and with every hop there is an inherent threat of packet loss due to a variety of reasons like channel errors, collisions, and dead or sleeping nodes [4]. Moreover, under this approach, a number of sensor nodes are acting as relaying nodes. Consequently, more energy is consumed on sensors which results in a shortened network lifetime.

### 4.4. Inter-Gateway Communication

The gateways are connected using high-speed Ethernet cables. Gateways are supposed to exchange certain information that would be required for routing and monitoring the state of the links. Our sensor network has been divided into clusters but the overall network can be considered as one autonomous system (AS). We therefore require a protocol configured in gateways that can handle cluster to cluster (Intra-AS) communication. We propose to employ the widely-used Open Shortest Path First (OSPF) protocol for inter-gateway communication. We choose OSPF because of two main reasons: (1) In distance vector routing protocols, each router does not possess information about the complete network topology and consequently there is a slow convergence problem (2) OSPF works over IP and has a richer set of extensions and added features as compared to other link state and distance vector routing protocols. Each gateway has information saved about nodes of the complete network in form of a gateway network table.

Every time a node is added or removed from a particular cluster, an update is sent to the default gateway. The default gateway accordingly updates the gateway network table. An update message is eventually sent to all the gateways of the network connected via the hotline. This completes one cycle of the update mechanism. This process is already supported in OSPF by the database synchronization mechanism. The synchronization process begins as soon as the gateway attempts to bring up the adjacency. The database is described by each gateway by sending a series of Database Description packets to its neighbors. As gateways are connected in a bus network, via hotline, all the gateways will synchronize their databases. Gateways are also deployed with a monitoring mechanism. Specifically, gateways are aware of the health of neighboring gateways and the corresponding links. This monitoring mechanism helps to ensure the connectivity of the gateways and eventually ensuring the availability of the hotline. This mechanism is supported by the OSPF Hello protocol. The neighbor relationship is maintained with the help of the Hello Protocol. It is also responsible of ensuring the bidirectional communication between neighbors. Hello packets are sent periodically on all router interfaces. In summary, gateways are configured with dual functionality. They are deployed with two different protocols. One protocol (WEAMR) is used for intra-cluster or cluster-node communication, the other protocol (OSPF) is used for inter-gateway communication.

### 4.5. Multipath Discovery Process

The multipath discovery process of WEAMR is based on AODV's route discovery process [1]. Please note that, our topology is cluster-based and the gateway acts as the cluster head. All the gateways are connected via high speed hotlines. Therefore, in the path discovery process the sender is actually finding a path to the gateway (intra-cluster communication). In other words, the communication pattern would be sender-gateway-gateway-destination. The destination gateway is most likely to be aware of the destination node, if not; the destination gateway would trigger the path discovery for the destination.

We have slightly modified the route discovery process. The format of the proposed RREQ message is shown in Figure 3. When a node needs to communicate with another node it first checks its routing table; if it does not find the destination it initiates the route discovery process. The node broadcasts the RREQ message containing an additional field of its energy level. This energy level will be used later to assess the quality of the path. Each RREQ packet has a unique identifier that is used by the intermediate nodes to detect and drop the duplicate RREQ messages. The destination sequence number in the RREQ message is used to find out the freshness of the packet. Upon receiving the RREQ message the intermediate node checks its routing table to see if it is familiar with the destination, and if it has the route to the destination it will reply with the RREP message and set the energy value in RREP by comparing the energy value of route path with energy value in RREQ message. The lower of these is set in the RREP message. Otherwise, it needs to set the reverse path, by recording the address of the RREQ sender, in its routing table. Then the intermediate node rebroadcasts the RREQ. Please note that the energy value will represent the lowest value of the path. Once a gateway receives the RREQ message it checks with its routing table if it has the destination. If a match is not found, it forwards the request to the destination associated gateway via hotline. The destination associated gateway would most likely know the destination and would already have established the multipath with the destination node, so it would reply with a RREP message. Otherwise, it will rebroadcast RREQ message to its associated cluster.

RREQ messages carry the path's energy information as well. Once the destination, in this case the sender associated gateway node, will receive the RREQ messages it will calculate the cost of each path and will decide to choose two best paths depending upon the minimum cost. The cost is calculated based on three factors: hop count, energy level and latency. The latency value is calculated based upon the mechanism suggested in [5]. The cost calculation is shown later in the equation.

**Figure 4.**
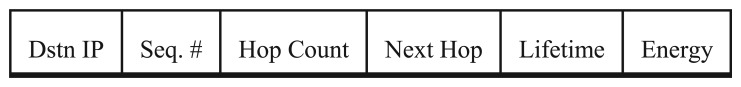
Proposed RREP message format.

Please note that only the destination node and the gateway nodes do not discard the duplicate RREQ messages. Once the destination receives the RREQ message, it waits for at least one more RREQ message to be received from the neighboring nodes. It then analyzes the RREQ messages and calculates and compares the cost. It then chooses two paths with minimum costs and unicasts the RREP message on the selected minimum cost paths. The proposed RREP message format is shown in Figure 4.

**Figure 5.**
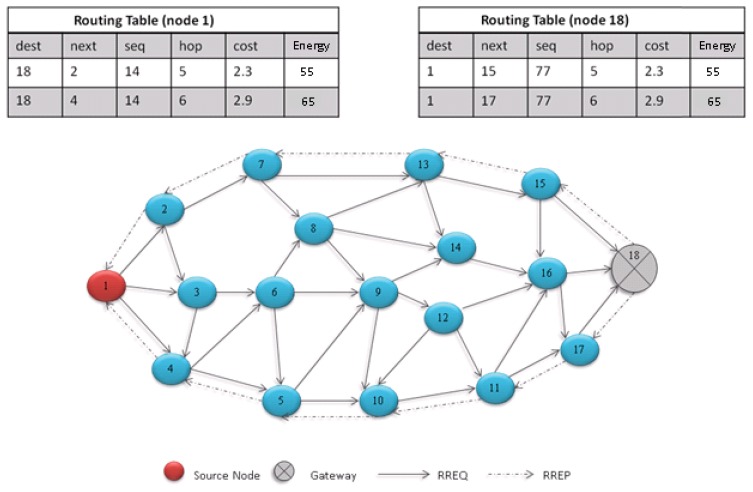
Overview of the multipath discovery process

The RREP message also contains the calculated cost. The source upon receiving the first RREP makes an entry in its routing table and for the second RREP message it makes another in its routing table along with the associated cost. The route with the minimum cost is used as the primary path whereas the other path is used as the backup path. Similarly, if the intermediate nodes receive two RREP messages it makes entries in to their routing tables. With this mechanism, the source has established two reliable paths with the destination. The process is shown in Figure 5. The cost calculation is given by the following equation:

$$C_{p}=\alpha H+\beta L+\frac{1}{\gamma^{E}}$$

where *C_P_* is the cost of the path, *H* represents the hop count, *L* represents the latency, *E* is the minimum energy of the path. *α*, *β* and *γ* are the coefficients that assign weight to the associated factors. Depending upon the requirement of the application, we can assign different weights to these factors. The value of these coefficients varies from 0 to 1. Moreover, hop count and the latency values are directly proportional to the path cost, whereas the energy value is inversely proportional to the path cost. WEAMR selects two best paths discovered based on the lowest calculated cost. In case of less dense networks where packet loss and latency is usually less we found out that hop *α* and latency *β* weightages should be kept under 0.10 and the energy coefficient *γ* should be kept over 0.80 so paths with more energy are used more frequently. In case of densely populated clusters with 1,000 nodes we found out that setting hop *α* and latency *β* coefficients to 0.35 and energy coefficient *γ* at 0.30 was giving us better throughput and reliability.

### 4.6. Data Transmission

Once the primary and secondary routes are established, the sender node would select the primary route and send its data. In order to balance the energy cost for the path the sender will switch between the discovered paths using a random approach. Although the idea proposed by [27] can be implemented to increase the efficiency of the network further, for the sake of simplicity we have used random switching strategy for data transmissions. In order to update associated cost of a discovered path *C_p_*, we suppose that average energy cost of transmitting an average sized message from one node to next node is Msg_ec_ with every transmission of a message the cost of the path is adjusted locally in sender's routing table as:

$$C_{p'}=\alpha H+\beta L+\frac{1}{\gamma^{E'}},\;\text{where}\frac{1}{\gamma^{E'}}=\frac{1}{\gamma^{E}}-{\text{Msg}}_{\text{ec}}$$

As we are using fixed topology the hop count and latency would remain reasonably constant, the only changing variable in the equation is energy. This local update mechanism for cost at the sender node incurs much less processing overhead and no additional control packets are required for updating the cost of the path. The intermediate nodes in the path might be involved in sending/forwarding data to the sink nodes which will make our locally calculated *C_p_*_′_ invalid. Since *C_p_*_′_ represents the cost value of the path which is calculated based on the lowest energy node value in the path, so to keep the value of *C_p_*_′_ updated with the lowest energy value node in the path our scheme proposes that every intermediate node checks its local energy value with expected lowest energy value sent in Data Packet by source node. This expected lowest energy value is populated by the source node from the energy field in routing table *i.e.*, *LE_p_* is value of lowest energy of a node on primary path. This value in the data packet is compared by intermediate nodes as the data packet moves toward the destination. An intermediate node sends back an update message to source node to update its *LE_p_* value if difference of energy value at the intermediate node and *LE_p_* sent in the data packet is lower than a threshold value *i.e.*,:

$$LE_{p}-E_{i}>E_{t}$$

where *E_t_* is a constant value and can be adjusted based on studying the energy depletion patterns of the network over a given time. *E_i_* is energy value at the intermediate node and *LE_p_* is the lowest energy value sent by the sender in data packet. In the case where the difference is within the threshold range, the intermediate node forwards the data packet. If the difference crosses the threshold then the intermediate node updates the expected energy value as *E_i_*, assuming itself to be the least energy node in the path and forwards the data packet. After transmitting the data packet the intermediate node sends a RREP update message to the source node with an updated sequence number and sets Energy as *E_i_* Any subsequent intermediate node can also generate an update RREP message in case its energy is less than *E_i_* The threshold range *E_t_* of intermediate nodes can be set in multiples of Msg_ec_, *i.e.*, a node having an *E_t_* of 5 * Msg_ec_ means that node will only update the sender if its energy level is less than the expected energy value sent by the sender by a value of 5 * Msg_ec_ *i.e.*, Cost of energy required to send five average sized messages. The sender and the intermediate nodes in reverse path receive the update RREP message and update the corresponding routing entry's *LE_p_* value and update the path cost in the table. The threshold difference value *E_t_* ensures the RREP updates are kept to a minimum and local cost update formulae based on message transmission cost ensures no additional network traffic as the cost for the path is updated locally. In case a link is broken the RERR messages received at the sender would mark the entry as invalid. The sender will try the secondary path if valid, and start a path discovery process if required to replace the expired path with a new path.

**Figure 6.**
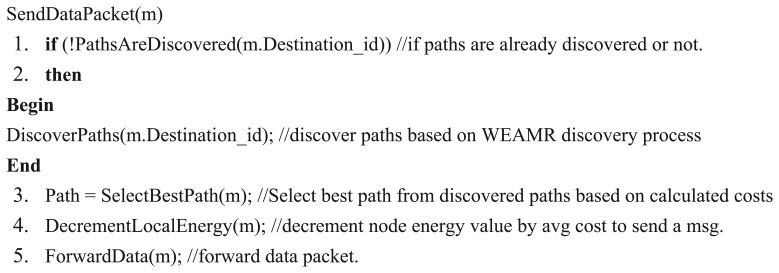
Algorithm to send data packet.

**Figure 7.**
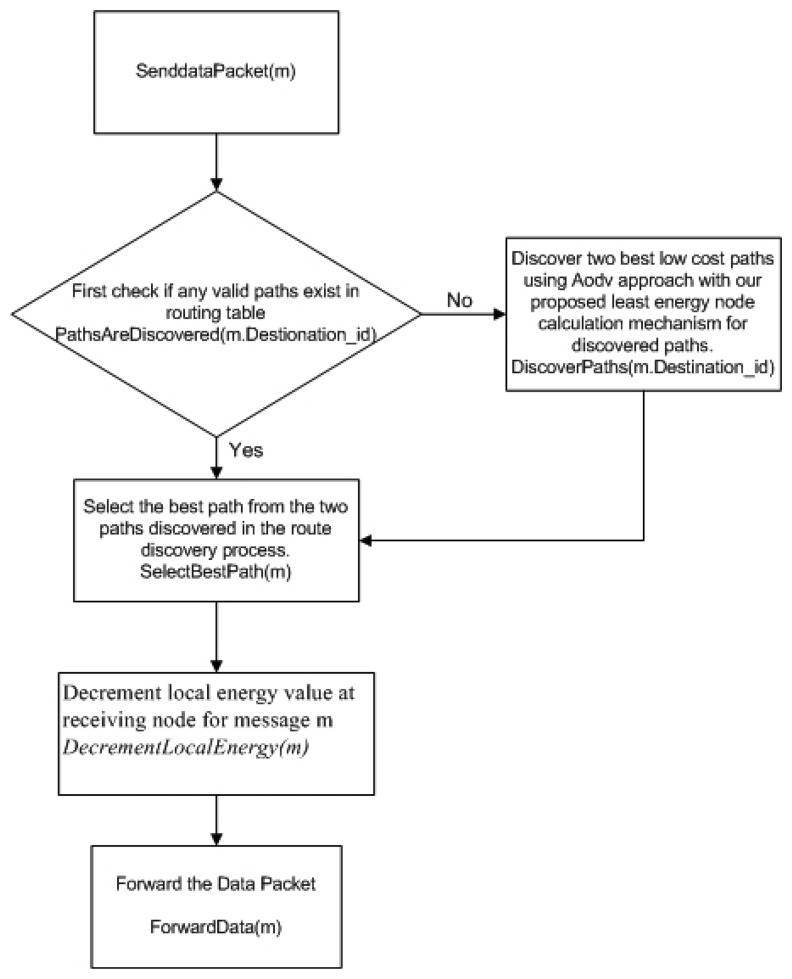
Overall procedure for the sender node to send data packet.

**Figure 8.**
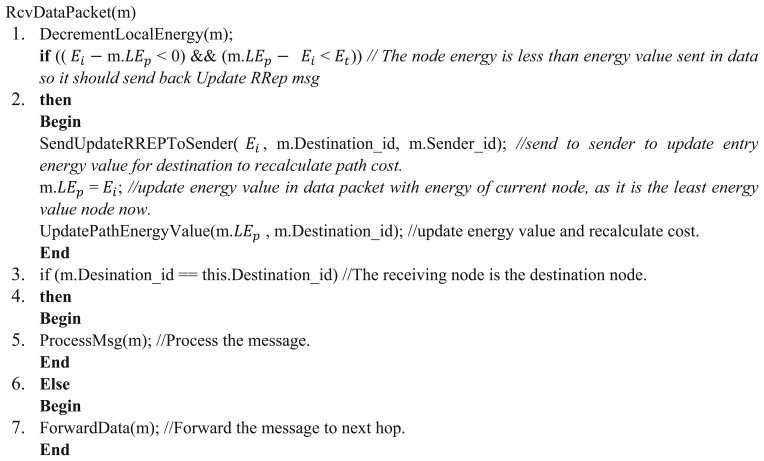
Algorithm to receive data packet.

**Figure 9.**
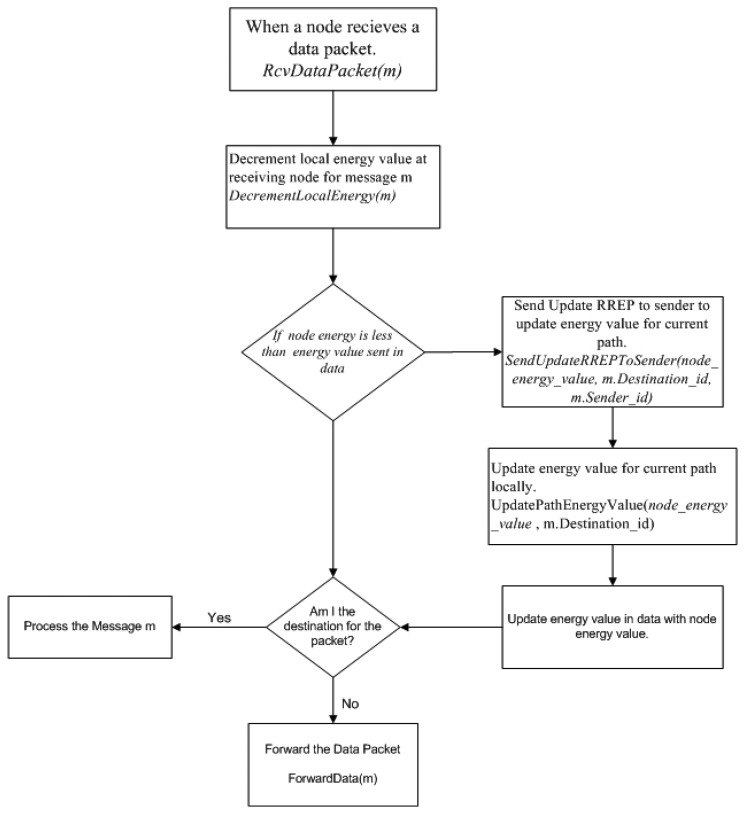
Overall procedure for intermediate node to receive data packet.

Figures 6, 7, 8 and 9 provide an insight to the overall mechanisms for the sender and the intermediate nodes to send data packets.

Since WEAMR is based on AOMDV so the path discovery method DiscoverPaths(m.Destination_id) takes equal amount of time as taken by AOMDV, The methods such as SelectBestPath(m), DecrementLocalEnergy(m) and ForwardData(m) complete their execution in linear time O(n). In case of delays or error the send timeout of 30 seconds ensures the execution is not delayed indefinitely for a process.

The methods DecrementLocalEnergy(m), SendUpdateRREPToSender(*E_i_*, m.Destination_id, m.Sender_id), UpdatePathEnergyValue(m.*LE_p_*, m.Destination_id) all execute in linear time O(n) in normal case.

## 5. Simulation

In this section we discuss the simulations and their results. Table 1 explains simulation setup and the environment that we utilized to conduct the simulations.

In hotline-assisted simulation scenarios the nodes are deployed in clusters as explained in Section 4.1. The cluster head act as a gateway and all the communication to and from the cluster takes place through the gateway. All the gateways are connected via Ethernet cable. Multiple sources send data to a sink to emulate bursty traffic. The protocols we have compared are AODV and AOMDV which do not handle hotline based routing natively. The factors that are used to measure the reliability of a WSN are: (1) Packet Success Ratio and (2) end-to-end Delay.

If the packets are being dropped frequently, that network will be considered unreliable. In some mission critical applications like military and healthcare immediate communication is required, therefore end-to-end Delay is also considered to be one of the important criterions for measuring the reliability [26]. We have divided the simulation discussion into three parts. Part A discusses the effect on reliability with respect to increasing number of connections from source to the sink, Part B talks about the effect on reliability with respect to increasing packet generation rate and in Part C we have compared the effect on reliability with respect to increasing node density.

### 5.1. Increasing Number of Connections

**Figure 10.**
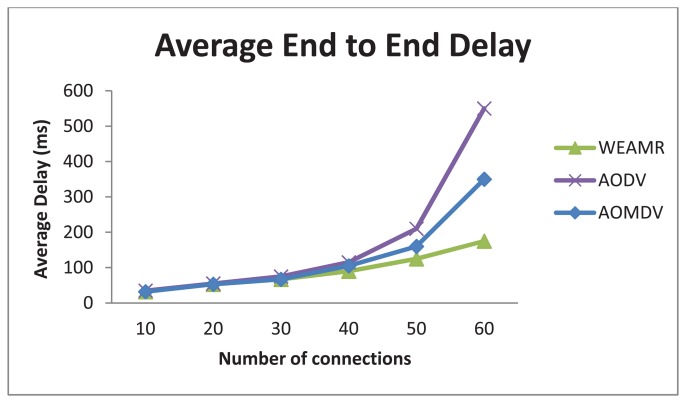
Average end to end delay with increasing number of connections.

**Figure 11.**
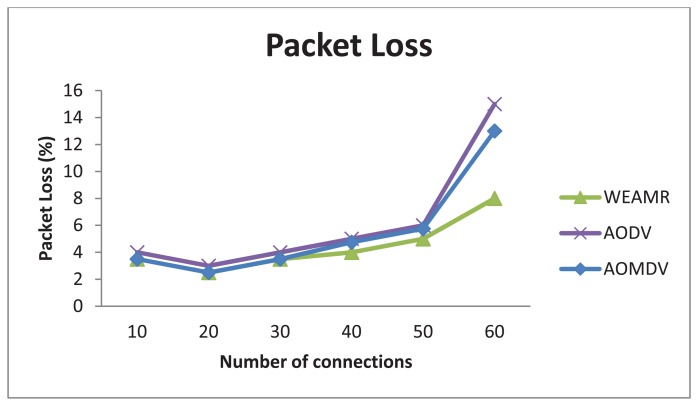
Average packet loss in end to end with increasing number of connections.

In this scenario we increased number of active connections in the network for data transfer. The number of nodes participating in this scenario was fixed to 10 nodes with varying number of connections per node. Each sending node was fixed with a total load of 30 MB of data and the transfer rate was initially set to 200 kb/s to produce congestion in the network. Each node started with one connection and once the data was transferred it increased the number of active connection by 1. The data load was divided in chunks according to the number of active connections on the sender node. This shows advantages of hotline-assisted routing in crowded networks. Moreover, each route discovery resulted in more expensive operation because of the smaller amount of traffic over each connection. The results shown in Figures 10 and 11 are the cumulative results of the simulations with regard to the number of connections between the source and the sinks.

#### 5.1.1. Average End to End Delay

As shown in Figure 10, WEAMR with our hotline-assisted approach outperforms both AODV and AOMDV as the number of connections increase. The average end to end delay increases with the increase in the number of simultaneous connections, but the average delay for AODV and AOMDV is much higher because these protocols work in hop by hop fashion for packet delivery and the hotline infrastructure is not used, whereas WEAMR shows lower end to end delay due to the fact that hotline-assisted routing can handle more simultaneous connections and reduce the number of intermediate hops.

#### 5.1.2. Average Packet Loss

As shown in Figure 11, WEAMR with hotline-assisted approach shows much more reliable results compared to purely *ad hoc*-based routing. We can notice that as the numbers of connections are increasing there is a decrease in the packet success ratio in both the cases, however, the packet drop rate is much higher in AODV and AOMDV as compared to WEAMR. One of the main reasons is that WSN nodes communicate in a wireless medium and with increasing number of hops the chances of packets loss increase exponentially. With a smaller number of connections, the difference between AODV and AOMDV is not very noticeable. However, as the number of connections increase, AOMDV performs better than legacy AODV due to the multipath use, but overall WEAMR outperforms AOMDV due to hotline-assisted routing which has better delivery throughput due to the use of a wired medium. Hotlines bypass erroneous wireless links and make every gateway just one hop away from all other gateways [26]. Therefore, the proposed topology enhances the packet success ratio manifolds as compared to traditional *ad hoc* wireless routing as shown in Figure 11.

#### 5.1.3. Route Discovery Frequency and Routing Overhead

**Figure 12.**
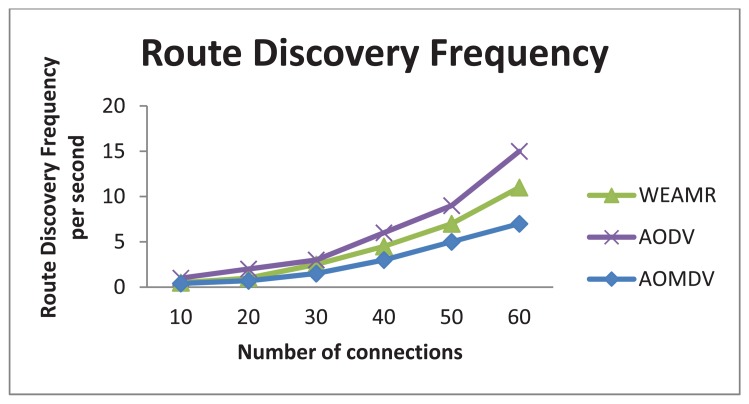
Average Route Discovery frequency with increasing number of connections.

In this case we calculated the average route discovery frequency of the three protocols per second. This is the number of times a protocol has to discover paths to the destination. The results shown here are intra-cluster-based discovery and does not include hotline assisted gateways, *i.e.*, The source and destination nodes are in same group of nodes. As shown in Figure 12, AOMDV has the minimum number of route discovery requests, and the reason for this is because AOMDV discovers all mutipaths available from source to destination, so when a path fails it does not have to rediscover, it simply uses the next available path. The disadvantage of this approach is overhead in route discovery process as it takes more time to discover all the paths available.

**Figure 13.**
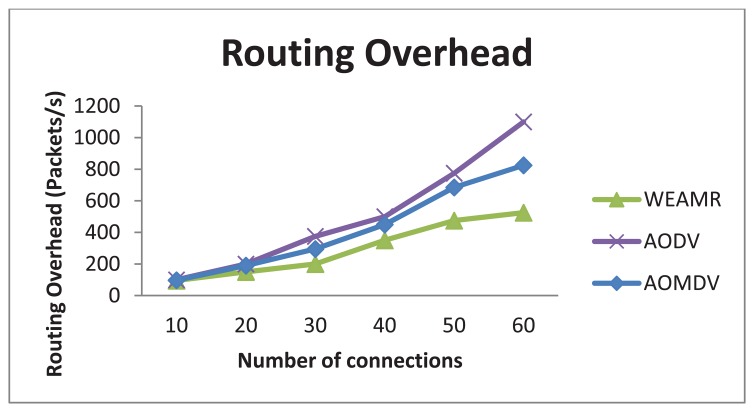
Average Routing overhead with increasing number of connections.

WEAMR resolves this issue by limiting the discoverable multipath value to two paths maximum. As discussed in [28], not all the paths discovered are suitable for routing and only minimum hop count paths contribute much to performance. Figure 12 shows result of route discovery frequency where WEAMR performs better than AODV. Figure 13 shows results for routing overhead of overall communication, in this simulation WEAMR shows significant less overhead due to less number of nodes involved in routing as hotline assisted routing saves a lot of hops in the transmission.

### 5.2. Varying Packet Generation Rate

In this scenario we have studied the effect of offered load and data rate on relative performance of the three protocols. The connections were kept constant at 5 connections. The packet generation data rate was increased from 0.25 to 1.50 packets/s to study the effect.

#### 5.2.1. Average End to End Delay

**Figure 14.**
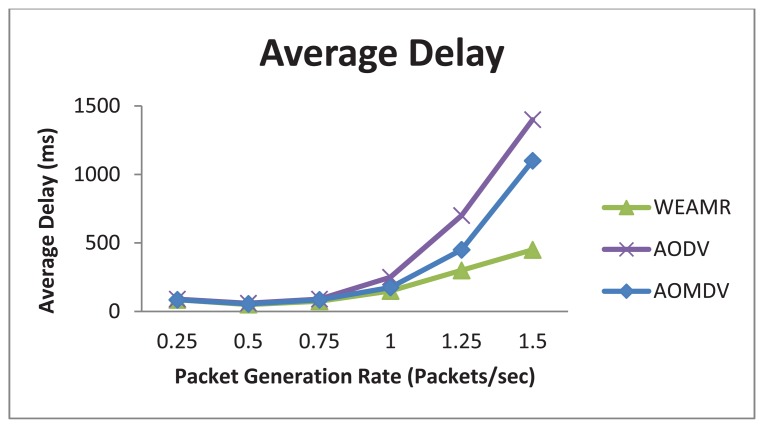
Average end to end delay with increasing number of packets.

As shown in Figure 14, WEAMR with hotline-assisted approach outperforms both AODV and AOMDV as connections are kept constant and the packet generation rate is increased. The average end to end delay increases with the increase in packet generation rate, however the average delay for AODV and AOMDV is much higher because these protocols work in hop by hop fashion for packet delivery and hotline infrastructure is not used. With very high packet rates performance for AODV and AOMDV reduces as they do not have any mechanism to mitigate congestion at high loads, whereas WEAMR shows lower end to end delay due to the fact that hotline-assisted routing can handle more simultaneous connections and reduce the number of intermediate hops.

#### 5.2.2. Average Packet Loss

**Figure 15.**
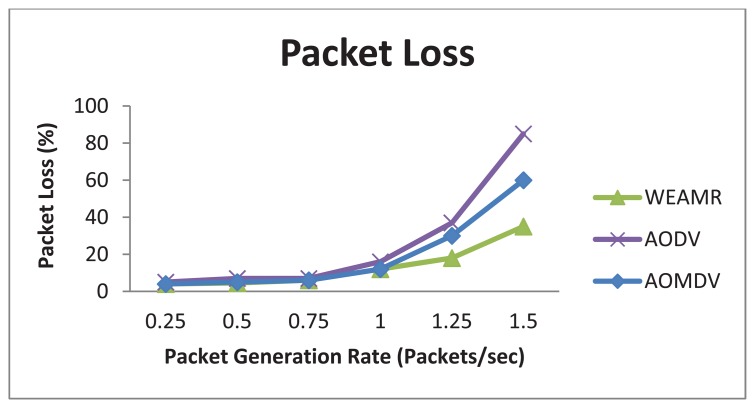
Average packet loss in end to end communication with increasing number of packets.

As shown in Figure 15, the average packet loss of WEAMR is lower than AODV and AOMDV. WEAMR is capable of handling a higher throughput hence the result for variable number of connections and variable packet rate are similar. Hotline topology enhances the packet success ratio manifolds as compared to traditional *ad hoc* wireless routing, as shown in Figure 15.

#### 5.2.3. Route Discovery Frequency and Routing Overhead

**Figure 16.**
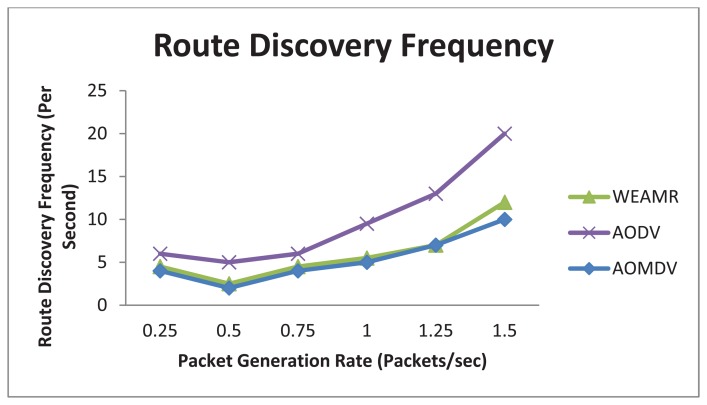
Average Route Discovery frequency with increasing number of packets.

In this case we calculated average route discovery frequency of the three protocols per second. This is the number of times a protocol has to discover route paths to the destination. The results shown here are intra-cluster-based discovery and does not include hotline assisted gateways. *i.e*., The source and destination nodes are in same group of nodes. As shown in Figure 16, AOMDV has minimum number of route discovery requests and WEAMR performs better than AODV.

**Figure 17.**
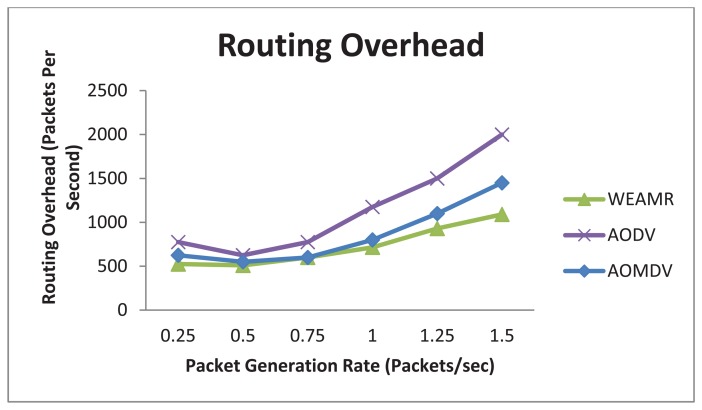
Average Routing overhead with increasing number of packets.

Figure 17 shows results for routing overhead of overall communication. In this simulation WEAMR shows less overhead due to less number of nodes involved in routing as hotline assisted routing saves a lot of hops in the transmission.

### 5.3. Varying Nodes Density in Network

In this scenario we have studied the effect of variable nodes density on relative performance of the three protocols. The active connections were kept constant at five connections. The packet generation data rate was also kept constant at 1.50 packets/s to study the effect. We started the simulation with a sparse network and then noticed the difference in reliability parameters with an increasing node density.

#### 5.3.1. Average End to End Delay

**Figure 18.**
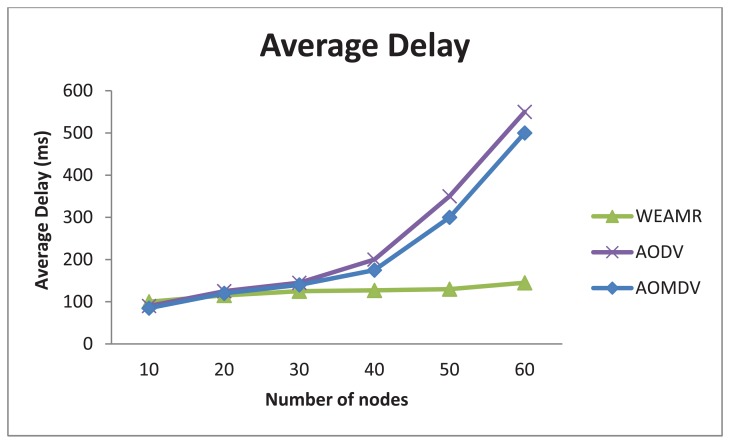
Average end to end delay with increasing number of nodes.

As shown in Figure 18, WEAMR with hotline-assisted approach outperforms both AODV and AOMDV as the number of connections and packet generation are kept constant and the nodes density in the clusters is increased linearly. Average end-to-end delay stays much lower and almost unchanged for WEAMR with the increasing number of nodes as compared to other protocols. AOMDV and AODV suffer from performance degradation because as node density increases the number of hops also increases, resulting in more communication overhead at each intermediate node, this increase in density the communication takes longer as compared to a less dense network [29], whereas for WEAMR the number of hops remain almost constant due to hotline assisted routing.

#### 5.3.2. Average Packet Loss

**Figure 19.**
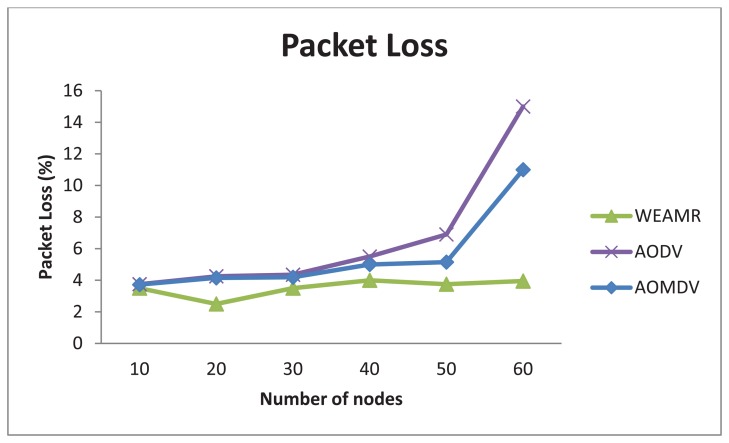
Average packet loss in end to end communication with increasing number of nodes.

As shown in Figure 19 below shows that WEAMR performs much better as compared to the AOMDV and AODV. As the number of nodes is increasing the packet loss average in the compared protocols is decreasing. This is because of the fact that with the increase in density of the network there will be more congestion and interference hence chances of packet loss will be higher [29]. On the other hand, we notice that packet success ratio for the hotline assisted routing remains almost unchanged regardless of the network density. The reason behind this is the permanent wired link between any given two gateways.

Therefore it will be fair to conclude that communication between the source and the sink is independent of the density of the network in the case of hotline assisted routing, so WEAMR also has lower routing overhead and routing path discovery requests when the node density is changing.

## 6. Conclusion

This paper proposes a new energy aware multipath routing protocol based on AODV and AOMDV in WSNs, which we have named as WEAMR. WEAMR makes use of a hotline-assisted routing approach which we verify can play an important role to improve the reliability of source to sink communications. WEAMR makes use of multipath transmission to utilize the network energy efficiently and keeps energy-aware related packet dissemination to a minimum, which results in longer lifetime of the network and also works as a load sharing feature. We showed through our simulation comparisons that WEAMR performs better than the AODV and AOMDV protocols in terms of inter-cluster end to end delay, has less packet loss in inter-cluster communication and has less route discovery overhead in case of intra-cluster path failure scenarios. The proposed topology provides reliability which is independent of the number of nodes in a given cluster or area. In the proposed protocol we have improved intra-cluster reliability and the lifetime of the network. Such features are very important for high density WSN deployments and these features make our routing protocol an ideal fit for all mission-critical applications.

## Figures and Tables

**Table 1.** Simulation setup.

Simulation Environment	NS3
Routing Protocol	WEAMR
Inter-Gateway Routing	OSPF
Intra-Cluster Communication	Wireless (802.15.4)
Number of Nodes	1,000
Node mobility	0 m/sec
Total Terrain Area	1,000 m × 1000 m
Simulation Time	1,000 s
Packet Loss nodes to node	2%
Packet Loss gateway to gateway	0%
Total Runs	15

## References

[1] Perkins C.E, Belding-Royer E. *Ad Hoc* On-Demand Distance Vector (AODV) Routing.

[2] Park V.D, Corson M.S. TORA, A Highly Adaptive Distributed Routing Algorithm for Mobile Wireless Networks.

[3] Johnson D.B., Maltz D.A., Imielinski T., Korth H. (1996). Dynamic Source Routing in *Ad Hoc* Wireless Networks. Mobile Computing.

[4] Perkins C.E, Bhagwat P. Highly Dynamic Destination-Sequenced Distance-Vector Routing (DSDV) for Mobile Computers.

[5] Tufail A., Khayam S.A., Raza M.T., Ali A., Kim K.-H. (2010). An Enhanced Backbone-Assisted Reliable Framework for Wireless Sensor Networks. Sensors.

[6] Gallardo J.R., Gonzalez A., Villasenor-Gonzalez L., Sanchez J. Multipath Routing Using Generalized Load Sharing for Wireless Sensor Networks.

[7] Popa L., Raiciu C., Stoica I., Rosenblum D. Reducing Congestion Effects in Wireless Networks by Multipath Routing.

[8] Intanagonwiwat C., Govindan R., Estrin D. Directed Diffusion: A Scalable and Robust Communication Paradigm for Sensor Networks.

[9] Heinzelman W.R., Kulik J., Balakrishnan H. Adaptive Protocols for Information Dissemination in Wireless Sensor Networks.

[10] Braginsky D., Estrin D. Rumor Routing Algorithm for Sensor Networks.

[11] Ye F., Chen A., Lu S. A Scalable Solution to Minimum Cost Forwarding in Large Sensor Networks.

[12] Shah R.C., Rabaey J.M. Energy Aware Routing for Low Energy *Ad Hoc* Sensor Networks.

[13] Yuan Y., Chen H.M., Jia M. An Optimized *Ad-Hoc* On-Demand Multipath Distance Vector (AOMDV) Routing Protocol Communications.

[14] Xiang M., Shi W.R., Jiang J.C., Ying Z.H. (2010). Energy Efficient Clustering Algorithm for Maximizing Lifetime of Wireless Sensor Network. Int. J. Electron. Commun..

[15] Karl H. (2005). Protocols and Architectures for Wireless Sensor Networks.

[16] Jung S., Akbar A.H., Roh B., Kim K. Multiple Routers-based Architecture for IPv6-Based Wireless Sensor Networks (6LoWPANs).

[17] Youssef W., Younis M. Intelligent Estimation of Gateways Count for Reduced Data Latency in Wireless Sensor Networks.

[18] Bogdanov A., Maneva E., Riesenfeld S. Power-Aware Base Station Positioning for Sensor Networks.

[19] Basagni S., Elia M., Ghosh R. ViBES: Virtual Backbone for Energy Saving in Wireless Sensor Networks.

[20] Stefano B., Chiara P., Roberto P. (2008). Efficiently Reconfigurable Backbones for Wireless Sensor Networks. Comput. Commun..

[21] Shin I., Kim M., Mutka M.W., Choo H., Lee T. (2009). MCBT: Multi-Hop Cluster Based Stable Backbone Trees for Data Collection and Dissemination in WSNs. Sensors.

[22] Hosam M.F., Iyengar S.S., Krishnendu C. (2005). Computing Reliability and Message Delay for Cooperative Wireless Distributed Sensor Networks Subject to Random Failures. IEEE Trans. Reliab..

[23] AboElFotoh H.M.F., ElMallah E.S., Hassanein H.S. On the Reliability of Wireless Sensor Networks.

[24] Shrestha A., Liudong X., Hong L. Modeling and Evaluating the Reliability of Wireless Sensor Networks.

[25] Shrestha A., Liudong X., Hong L. Infrastructure Communication Reliability of Wireless Sensor Networks.

[26] Tufail A., Khayam S.A., Hwan S.D., Kim K.H. A Hotline-Based Reliable Topology for Wireless Sensor Networks.

[27] Li M., Zhang L., Li V. O. K., Shan X., Ren Y. An Energy-Aware Multipath Routing Protocol for Mobile *Ad Hoc* Networks.

[28] Nasipuri A., Castaneda R., Das S.R. (2001). Performance of Multipath Routing for On-Demand Protocols in Mobile *Ad Hoc* Networks. J. Mobile Netw. Appl..

[29] Mamun-Or-Rashid M., Muhammad M.A., Abdur R.M., Choong S.H. Reliable Event Detection and Congestion Avoidance in Wireless Sensor Networks.

